# Annual summaries dataset of Heatwaves in Europe, as defined by the Excess Heat Factor

**DOI:** 10.1016/j.dib.2022.108511

**Published:** 2022-08-04

**Authors:** Ana Oliveira, António Lopes, Ezequiel Correia

**Affiliations:** a IPL-ESTM, Rua do Conhecimento nº4 2520-614 Peniche, Portugal; b IN+ Center for Innovation, Technology and Policy Research, Instituto Superior Técnico, Universidade de Lisboa, Av. Rovisco Pais 1, Lisboa 1049-001, Portugal; c Centre of Geographical Studies, Institute of Geography and Spatial Planning, Edificio I, University of Lisbon, R. Branca Edmée Marques, Lisboa 1600-276, Portugal

**Keywords:** E-OBS, Heatwaves, Excess heat factor, Europe, Geodata, climate extremes

## Abstract

The dataset includes six yearly time series of six Heatwave (HW) aspects/metrics (or statistical summaries) calculated from the E-OBS dataset (v19eHOM, available in https://www.ecad.eu/download/ensembles/downloadversion19.0eHOM.php) following the Excess Heat Factor (EHF) methodology implemented in the ClimPACT tool, in compliance with the guidelines established by the Expert Team on Climate Change Detection and Indices (ET-SCI). These aspects correspond to annual summaries of HW frequency, duration and intensity, considering solely the events occurring during the extended summer season (from June to September). Input Daily Maximum (TX) and Minimum (TN) near-surface air temperature data were retrieved from a European gridded dataset (E-OBS) – the ensemble homogenized version ‘19.0eHOM’, at 0.1° × 0.1° spatial resolution, covering the European region, and retrieved from the EU-FP6 project UERRA (http://www.uerra.eu) and the Copernicus Climate Change Service. The E-OBS dataset is based on station observations, provided by the European Climate Assessment & Dataset. The here-presented HW aspects/summaries outputs of the ClimPACT tool correspond to the gridded annual statistical summaries of HW – these are detected based on the positive Excess Heat Factor (EHF) days, an HW index based on the human health response to heat extremes. The summaries include: (i) annual Number of Heatwaves (HWN); (ii) annual Heatwave Days Frequency (HWF); (iii) annual Maximum Heatwave Duration (HWD); (iv) annual Mean Heatwave Magnitude (HWM); and (v) annual Maximum Heatwave Amplitude (HWA). In addition, the annual maximum Heatwave Severity (HWS) was calculated, by dividing HWA by the 85th percentile of the positive EHF days. These annual time series can be used in HW-related studies focusing on the European region, particularly those focusing on climatology, trends, and impacts on human health.

## Graphical abstract

**Figure:**
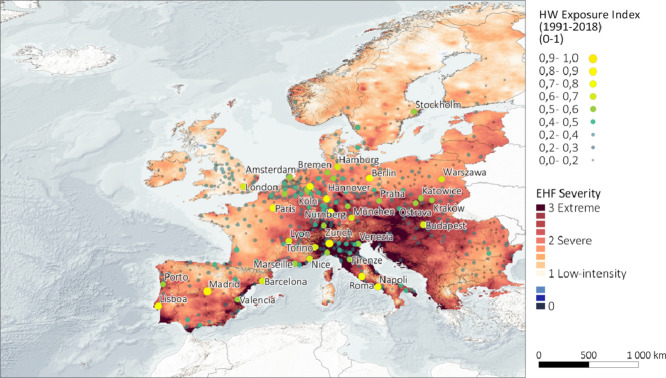

**Specifications Table**

**Table:** 

Subject	Environmental Science (Climatology)
Specific subject area	Observed atmospheric heatwaves in Europe, from 1950 to 2018. Annual summaries of heatwave aspects/metrics, following the Excess Heat Factor (EHF) methodology
Type of data	GeoTIFF format EHF-based heatwave aspects/metrics
How the data were acquired	The input datasets correspond to gridded Daily Maximum (TX) and Minimum (TN) near-surface air temperature values, based on stations observations, subsetted to the extended summer season (from June to September). These were retrieved from the gridded Europe-wide ensemble dataset (E-OBS) - homogenized version ‘19.0eHOM’, at 0.1° × 0.1° spatial resolution. The input data was retrieved from the EU-FP6 project UERRA (http://www.uerra.eu) and the Copernicus Climate Change Service, based on station observations data provided by the European Climate Assessment & Dataset (ECA&D project, available at https://www.ecad.eu). EHF-based heatwave aspects/metrics were computed from these data using the ClimPACT R-based tool (https://github.com/ARCCSS-extremes/climpact/)
Data format	Analyzed
Description of data collection	The input air temperature data was retrieved from the ensemble dataset (E-OBS) - homogenized version ‘19.0eHOM’, at 0.1° × 0.1° spatial resolution. This data source includes daily TX and TN ‘best guess’ estimations (mean) from an ensemble of 100 members covering the 1950 2018 period. The 19.0eHOM version results from the interpolation of station-derived meteorological observations from the ECA&D initiative, following quality control, including homogeneity tests and corrections. Pre-processing was done with Climate Data Operators (CDO), to comply with ClimPACT instructions. Following the guidelines from the ClimPACT2 tool, the EHF calculation on gridded data is done in a Linux-based environment, and its output comprises annual HW time series, depicting the most frequently used HW aspects/metrics (i.e., annual statistical summaries): (i) annual Number of Heatwaves (HWN); (ii) annual Heatwave Days Frequency (HWF); (iii) annual Maximum Heatwave Duration (HWD); (iv) annual Mean Heatwave Magnitude (HWM); and (v) annual Maximum Heatwave Amplitude (HWA). Since the HWA/HWM annual values can only be compared meaningfully after HW intensity normalization, the annual maximum EHF-based severity metric (HWS) has been added by the authors – this was done by computing the annual time series of the normalized HWA index per its 85th percentile. The reference climatology was the 1961–1990 period, considering the extended summer period (from June to September).
Data source location	Primary data source: access and metadata available from the ECA&D initiative website (https://www.ecad.eu/). Type of data: regular grid of elements Original format: NetCDF-4 Latitude and longitude spatial coverage: 25N-71.5 N x 25W-45E Spatial Resolution: 0.1 × 0.1° Temporal coverage: from 1 January 1950 to 31 December 2018 Frequency: daily Variables: maximum and minimum temperatures (TX and TN, respectively)
Data accessibility	Public repository Repository name: Mendeley data Data identification number: 10.17632/n33fzh6wgy.1 Direct URL to data: http://dx.doi.org/10.17632/n33fzh6wgy.1
Related research article	A. Oliveira, A. Lopes, A. Soares, Excess Heat Factor climatology, trends, and exposure across European Functional Urban Areas, Weather and Climate Extremes. 36 (2022) 100,455. 10.1016/j.wace.2022.100455

**Value of the Data**

• Atmospheric Heatwaves (HW) are known to have significant impacts on human health, for example, triggering excess mortality episodes. In particular, the Excess Heat Factor (EHF) index has been shown to have significant advantage in predicting these impacts. Accordingly, the datasets here provided correspond to six aspects/metrics derived from the EHF index, which can be used to investigate the annual variability and trends in HW frequency, duration, intensity, and its relationship with other climate change prospects.
• Researchers interested in the climate and health domains may benefit from this data. In addition, stakeholders involved in climate risk assessment may also use the data provided.
• From the research point of view, the data allows for recognizing recent HW occurrence patterns, as well as correlating those with other atmospheric processes (e.g., climate modes, leading atmospheric events) or impacts (e.g., human health, healthcare demand). In addition, the Heatwave metrics can also provide insights for risk assessment and climate adaptation.

## 1 Data Description

The Excess Heat Factor (EHF) is an atmospheric Heatwave (HW) index developed with the aim of establishing a meaningful proxy to estimate the human health impacts deriving from extreme heat temperatures exposure [1]. The dataset here provided [2] is based on this EHF algorithm, and includes six annual time series, each depicting the HW summary statistics (also known as aspects/metrics) from 1950 up to 2018, considering HW occurring between June and September each year, in the European region. The EHF metrics were calculated from the E-OBS dataset (v19eHOM, available at https://www.ecad.eu/) – this is an observation-based gridded meteorological data source that results from the interpolation of in-situ measurements that are subject to quality control, including homogeneity tests and corrections [3,4], and available from the ECA&D initiative [5,6]. Pre-processing of the original E-OBS TX and TN datasets was done with Climate Data Operators (CDO), to comply with ClimPACT instructions. The EHF calculation was implemented in R processing language [7], following the ClimPACT tool [8] guidelines, which were developed by one of the specialized teams endorsed by the World Meteorological Organization (WMO) – the Expert Team on Sector-Specific Climate Indices (ET-SCI).

Following the framework of the ClimPACT tool, the results of the EHF computation are gathered as annual HW time series, depicting the five so-called HW aspects/metrics that correspond to the annual statistical summaries (e.g., mean, sum, maximum) most used in climatology studies to highlight the frequency, duration and intensity of climate extremes. In addition, the annual maximum Heatwave Severity (HWS) was calculated – the motivation to add this sixth metric is based on the fact that EHF is a percentile-based index, hence, its intensity is strongly biased by the local-specific temperature variability. Accordingly, for comparison purposes, a normalised version of the absolute EHF intensity must be conducted, by dividing HWA by the 85th percentile of the positive EHF days - details regarding HWS trends obtained from this dataset are described in the source scientific publication [9], and follows the rationale depicted in a previous study [10].

Following these considerations, each geodata file here provided (in GeoTIFF format, 0.1 × 0.1° spatial resolution, EPSG:4326 projection) depicts an historical and observations-based time series of a given type of HW annual statistical summary, comprising 69 bands each corresponding to one year, covering the 1950 to 2018 period. These HW annual aspects/metrics can be described as in the following Table 1, Table 2, Table 3, Table 4, Table 5, Table 6.

**Table 1:** Metadata for the Annual Number of Heatwaves (HWN) time series.

Dataset Name:	Annual Number of Heatwaves (HWN)
Filename:	ehf_eobs_e19HOM_1950–2018_reference_1961–1990_ann_hwn.tif
Description:	Corresponds to the annual sum of the number of HW events, i.e., the number of EHF-positive instances of consecutive days, considering that a minimum of 3-days positive deviation from the 90th percentile must exist for a positive EHF to be detected. This is a frequency metric, and covers the HW events in Europe, considering the extended summer season (June-September), from 1950 to 2018, in relation to the 1961–1990 climatology.
CRS:	WGS84 EPSG:4326
Extent:	−180.0, −21.3; +189.0, +82.0
Data type:	Floating point, 32 bits
Format:	Geotiff
Dimensions:	X (longitude): 3599 (degrees east) Y (latitude): 1031 (degrees north) Bands (time): 69 (years since 1949)
Pixel size:	0.1 × 0.1 (degrees)
Variable:	Number of HW events
Unit:	Events
Missing value:	−999
Data source:	E-OBS, v19.0eHOM [11]
Data source type:	Meteorological observations
Calculation method:	ClimPACT2 [8]

**Table 2:** Metadata for the Heatwave Days Frequency (HWF) time series.

Dataset Name:	annual Heatwave Days Frequency (HWF)
Filename:	ehf_eobs_e19HOM_1950–2018_reference_1961–1990_ann_hwf.tif
Description:	Corresponds to the annual sum of the number of HW days, i.e., the total amount of EHF-positive days, in which the 90th percentile for a given day of the year (DOY) is surpassed. This is a frequency metric, and covers the HW events in Europe, considering the extended summer season (June-September), from 1950 to 2018, in relation to the 1961–1990 climatology.
CRS:	WGS84 EPSG:4326
Extent:	−180.0 (latitude), −21.3(longitude); +189.0 (latitude), +82.0 (longitude)
Data type:	Floating point, 32 bits
Format:	Geotiff
Dimensions:	X (longitude): 3599 (degrees east) Y (latitude): 1031 (degrees north) Bands (time): 69 (years since 1949)
Pixel size:	0.1 × 0.1 (degrees)
Variable:	Number of HW days
Unit:	Days
Missing value:	−999
Data source:	E-OBS, v19.0eHOM [11]
Data source type:	Meteorological observations
Calculation method:	ClimPACT2 [8]

**Table 3:** Metadata for the Annual Maximum Heatwave Duration (HWD) time series.

Dataset Name:	annual Maximum Heatwave Duration (HWD)
Filename:	ehf_eobs_e19HOM_1950–2018_reference_1961–1990_ann_hwd.tif
Description:	Corresponds to the annual maximum HW length (in number of days), i.e., the total amount of EHF-positive days of the longest HW of each year (days in which the 90th percentile for a given day of the year (DOY) is surpassed). This is a duration metric, and covers the HW events in Europe, considering the extended summer season (June-September), from 1950 to 2018, in relation to the 1961–1990 climatology.
CRS:	WGS84 EPSG:4326
Extent:	−180.0 (latitude), −21.3(longitude); +189.0 (latitude), +82.0 (longitude)
Data type:	Floating point, 32 bits
Format:	Geotiff
Dimensions:	X (longitude): 3599 (degrees east) Y (latitude): 1031 (degrees north) Bands (time): 69 (years since 1949)
Pixel size:	0.1 × 0.1 (degrees)
Variable:	Number of HW days
Unit:	Days
Missing value:	−999
Data source:	E-OBS, v19.0eHOM [11]
Data source type:	Meteorological observations
Calculation method:	ClimPACT2 [8]

**Table 4:** Metadata for the Annual Mean Heatwave Magnitude (HWM) time series.

Dataset Name:	annual Mean Heatwave Magnitude (HWM)
Filename:	ehf_eobs_e19HOM_1950–2018_reference_1961–1990_ann_hwm.tif
Description:	Corresponds to the annual EHF mean intensity (in °C^2^), i.e., the average quadratic temperature anomaly as a function of the long-term (i.e., from the 90th percentile for a given day of the year (DOY)) and short-term (i.e., from the last 30 days) deviations. This is an intensity metric, and covers the HW events in Europe, considering the extended summer season (June-September), from 1950 to 2018, in relation to the 1961–1990 climatology.
CRS:	WGS84 EPSG:4326
Extent:	−180.0 (latitude), −21.3(longitude); +189.0 (latitude), +82.0 (longitude)
Data type:	Floating point, 32 bits
Format:	Geotiff
Dimensions:	X (longitude): 3599 (degrees east) Y (latitude): 1031 (degrees north) Bands (time): 69 (years since 1949)
Pixel size:	0.1 × 0.1 (degrees)
Variable:	Mean EHF intensity
U nit:	°C^2^_L_
Missing value:	−999
Data source:	E-OBS, v19.0eHOM [11]
Data source type:	Meteorological observations
Calculation method:	ClimPACT2 [8]

**Table 5:** Metadata for Annual Maximum Heatwave Amplitude (HWA) the time series.

Dataset Name:	annual Maximum Heatwave Amplitude (HWA)
Filename:	ehf_eobs_e19HOM_1950–2018_reference_1961–1990_ann_hwa.tif
Description:	Corresponds to the annual EHF maximum intensity (in °C^2^), i.e., the maximum quadratic temperature anomaly as a function of the long-term (i.e., from the 90th percentile for a given day of the year (DOY)) and short-term (i.e., from the last 30 days) deviations. This is an intensity metric, and covers the HW events in Europe, considering the extended summer season (June-September), from 1950 to 2018, in relation to the 1961–1990 climatology.
CRS:	WGS84 EPSG:4326
Extent:	−180.0 (latitude), −21.3(longitude); +189.0 (latitude), +82.0 (longitude)
Data type:	Floating point, 32 bits
Format:	Geotiff
Dimensions:	X (longitude): 3599 (degrees east) Y (latitude): 1031 (degrees north) Bands (time): 69 (years since 1949)
Pixel size:	0.1 × 0.1 (degrees)
Variable:	Maximum EHF intensity
U nit:	°C^2^_L_
Missing value:	−999
Data source:	E-OBS, v19.0eHOM [11]
Data source type:	Meteorological observations
Calculation method:	ClimPACT2 [8]

**Table 6:** Metadata for Annual Maximum Heatwave Severity (HWS) the time series.

Dataset Name:	annual Maximum Heatwave Severity (HWS)
Filename:	ehf_eobs_e19HOM_1950–2018_reference_1961–1990_ann_hws.tif
Description:	Corresponds to the annual maximum EHF severity, i.e., the maximum EHF anomaly in each year (HWA) divided by the 85th percentile of positive EHF intensities from the climatological reference period. This is a severity metric, and covers the HW events in Europe, considering the extended summer season (June-September), from 1950 to 2018, in relation to the 1961–1990 climatology.
CRS:	WGS84 EPSG:4326
Extent:	−180.0 (latitude), −21.3(longitude); +189.0 (latitude), +82.0 (longitude)
Data type:	Floating point, 64 bits
Format:	Geotiff
Dimensions:	X (longitude): 3599 (degrees east) Y (latitude): 1031 (degrees north) Bands (time): 69 (years since 1949)
Pixel size:	0.1 × 0.1 (degrees)
Variable:	Maximum EHF severity
Unit:	Dimensionless (normalized)
Missing value:	−999
Data source:	E-OBS, v19.0eHOM [11]
Data source type:	Meteorological observations
Calculation method:	ClimPACT2 [8]

## 2 Experimental Design, Materials and Methods

The original source data is corresponds to a gridded dataset containing interpolations of station-derived meteorological observations and was retrieved from the ECA&D website (available at https://www.ecad.eu/). The version used is the ensemble homogenized dataset, ‘E-OBS 19.0eHOM’, at a 0.1° × 0.1° spatial resolution. The original dataset includes daily maximum and minimum air temperature ‘best guess’ estimations (mean) of 100 ensemble members (TX and TN, respectively), covering a 69-year period, from 1950 to 2018 [11]. The 19.0eHOM version follows several quality control procedures, including homogeneity tests and corrections [11]. Pre-processing of the original E-OBS data (in NetCDF) was done using the Climate Data Operators (CDO) [12], to comply with ClimPACT2 instructions [13], namely merging the original TX and TN into a single file and renaming the variables. The HW aspects/metrics were computed in the ClimPACT2 R-based tool - the tools’ calculation method follows existing literature [14] which defines the EHF as the product of two excess heat sub-indices: (i) the Excess Heat Index Significance (EHI_sig_), which depicts the long-term (i.e., climatological) anomaly, by measuring the difference between the 3-days daily mean temperature (TM) and the equivalent 90th percentile; and (ii) the Excess Heat Index Acclimatization (EHI_accl_) which depicts the short-term anomaly, by measuring the difference between the last 3-days TM and that of the preceding 30 days. The 90th percentile is calculated per day of the year (DOY) for the user-specified reference period. Here the 1961–1990 30-year period is used as the reference climatology, and the DOY percentiles are smoothed by running a 15-days rolling average window. The two sub-indices and the EHF index are calculated according to Eqs. (1)–(3):

(1) $$\text{EH}I_{\text{sig}}=\;TM_{3-\text{day}}-\;TM_{90p}$$

(2) $$\text{EH}I_{\text{accl}}=\;TM_{3-\text{day}}-\;TM_{30-\text{day}}$$

(3) $$\text{EHF}\;=\;\text{EH}I_{\text{sig}}\;\times \text{MAX}\left(1,\;\text{EH}I_{\text{accl}}\right)$$

where TM_3-day_ (TM_30-day_) is average TM over 3 (30) days, respectively (calculated as the average between TN and TX), and TM90p is the 90th percentile of TM for the correspondent calendar DOY. Calculations were done per each grid cell of the E-OBS input data (i.e., pixel-wise calculation). For an HW event detection, a minimum of 3 consecutive days with positive EHF has to occur. In such cases, the EHF pixel-wise value represents a quadratic measure of the HW intensity. It is measured in °C^2^_L_, where the ‘_L_’ subscript highlights the fact that intensities are local-specific – i.e., lower values are expected where the climate temperature range is lower [10].

The output of the ClimPACT2 tool provides EHF-based statistical summaries, gathered as an annual HW time series, each depicting one of five HW aspects/metrics mentioned in the previous section: (i) annual Number of Heatwaves (HWN); (ii) annual Heatwave Days Frequency (HWF); (iii) annual Maximum Heatwave Duration (HWD); (iv) annual Mean Heatwave Magnitude (HWM); and (v) annual Maximum Heatwave Amplitude (HWA). As previously mentioned, the two HW intensity summaries (i.e., HWA and HWM) are only meaningful for a given local temperature range, and a normalization procedure is required to compute a comparable HW intensity metric. To this effect, the HWA results were normalized by the 85th percentile of EHF intensities (EHF85p) over the same 1961–1990 reference climatology period (see Eq. (4)) – the results is a time series of the annual Maximum Heatwave Severity (HWS) values (normalized/dimensionless).

(4) $$\text{HWS}\;=\;\text{HWA}\;÷\;\text{EH}F_{85p}$$

All the data processing and calculations were run on a local personal computer (PC) laptop, in a Linux-based operating system (CPU: Intel® Core™ i7–9750H, 2.60 GHz, RAM: 16.0GB, system type: 64-bit, Memory: 15.9 GB; GPU: NVIDIA GeForce GTX 1650). Data visualization was conducted in the open-source geographic information system software QGIS [15].

## CRediT authorship contribution statement

**Ana Oliveira:** Conceptualization, Methodology, Investigation, Data curation, Writing – original draft, Writing – review & editing. **António Lopes:** Conceptualization, Methodology, Writing – review & editing. **Ezequiel Correia:** Conceptualization, Methodology, Writing – review & editing.

## Declaration of Competing Interest

The authors declare that they have no known competing financial interests or personal relationships that could have appeared to influence the work reported in this paper.

## Data Availability

Annual Summaries of Heatwave Aspects in Europe, as defined by the Excess Heat Factor (Original data) (Mendeley Data).
